# Use of Next-Generation Sequencing for Diagnosis of West Nile Virus Infection in Patient Returning to Belgium from Hungary 

**DOI:** 10.3201/eid2412.180494

**Published:** 2018-12

**Authors:** Elke Wollants, David Smolders, Reinout Naesens, Peggy Bruynseels, Katrien Lagrou, Jelle Matthijnssens, Marc Van Ranst

**Affiliations:** KU Leuven Rega Institute, Rega Institute, Leuven, Belgium (E. Wollants, J. Matthijnssens, M. Van Ranst);; ZNA Hospital Middelheim, Antwerp, Belgium (D. Smolders, R. Naesens, P. Bruynseels);; University Hospital Leuven, Leuven, Belgium (K. Lagrou, M. Van Ranst)

**Keywords:** complete genome, meningitis/encephalitis, next-generation sequencing, respiratory culture, vector-borne infections, viruses, West Nile virus, Belgium, Hungary, respiratory infections

## Abstract

An elderly patient in Belgium who became critically ill after returning from Hungary was tested for pathogens using routine diagnostic tests. All results were negative. However, using next-generation sequencing on a cultured respiratory sample, laboratorians detected a complete West Nile virus genome, similar to strains isolated in southeastern Europe.

West Nile virus (WNV) is a widespread reemerging global pathogen. Mosquito bites are the source for nearly all human infections. These infections usually remain asymptomatic; only 20%–40% of infected persons experience a mild influenza-like illness called West Nile fever. West Nile neuroinvasive disease, which can manifest as meningitis, encephalitis, or acute flaccid paralysis, develops in 1% of infected persons; immunosuppressed and elderly patients are at higher risk (*1*). WNV can be divided into 5 phylogenetic lineages, of which lineages 1 and 2 have been associated with many outbreaks in humans. Since 2010, occasional localized epidemics have been ongoing in European and Mediterranean countries (*2*). To date, no autochthonous WNV infections have been reported in Belgium, and imported cases are uncommon (*3*,*4*). 

An 83-year-old man residing in Antwerp, Belgium, who had a medical history of moderate chronic kidney disease traveled to Hungary from January through August 2017. One day after returning from Hungary, he went to the hospital emergency department. He had fever and sought treatment for pain in his mouth and throat, which had begun the day before. He reported having felt unusually fatigued over several days. Clinical examination showed the patient to be lethargic but having no abnormal neurologic signs. 

Laboratory testing at admission revealed a normal leukocyte count of 6.8 × 10^9^ cells/L and a moderately elevated C-reactive protein level of 63.2 mg/L. A urine sediment test, a chest radiograph, and an abdominal ultrasound revealed no signs of disease. Treatment with amoxicillin/clavulanate was started, and blood and urine samples for cultures were collected. After 5 days, the patient was transferred to the intensive care unit because of increasing lethargy, followed by loss of consciousness, and clinical and biochemical signs of multiple organ failure. He was intubated and his treatment changed to piperacillin/tazobactam and clarithromycin; new cultures were also performed. Fever persisted, and the patient developed pancytopenia (leukocytes 1.68 × 10^9^ cells/L; hemoglobin 8.6 g/dL; platelets 61 × 10^9^/L). Because kidney function deteriorated further, continuous venovenous ultrafiltration hemodialysis was begun. An electroencephalogram exam showed delayed response without signs of epilepsy. A cranial computed tomography scan showed no signs of acute disease, and autoimmune screening did not reveal any abnormalities. 

The results from a multiplex PCR (RespiFinder22; PathoFinder, Maastricht, the Netherlands), performed on a broncheoalveolar lavage (BAL) sample, were negative for 22 different respiratory pathogens. The result from a singleplex PCR for *Mycobacterium tuberculosis* was negative. Test results from immunofluorescence and PCR for *Pneumocystis jirovecii* were also negative. Cerebrospinal fluid testing revealed an elevated pH of 7.95, elevated total protein of 79 mg/dL, normal glucose of 65 mg/dL, slightly increased leukocytosis of 7/mm^3^, and elevated erythrocytes of 370/mm^3^. Results were negative for molecular diagnostics of cerebrospinal fluid for 13 pathogens and serology testing for 12 pathogens. Results from viral cultures on a BAL sample, taken 8 days after hospital admission, showed a cytopathogenic effect on 3 available cell lines (Vero, HEp-2, and MRC5) 7 days after incubation. Results from a PCR, administered in an effort to identify the positive viral culture, for herpes simplex virus and enterovirus were both negative.

Because the general condition of the patient had improved and stabilized, he was weaned from intubation 14 days after admission to the intensive care unit. He was discharged to the geriatric department and slowly recovered consciousness; his neurologic status also normalized. Three months after onset of the infection, the patient described having some memory loss and no memory at all of the days when the infection first started.

Because routine testing did not lead to a diagnosis, a BAL sample and a viral culture were sent to the University Hospital Leuven (Leuven, Belgium). The cell culture was subjected to next-generation sequencing (NGS) with the novel enrichment technique of VIRomes protocol (*5*). This protocol includes purification of viral particles, nucleic acid extraction, and random PCR amplification, followed by library preparation and sequencing using NextSeq 500 system NGS technology (Illumina, San Diego, CA, USA) (*6*). The sequence reads obtained were filtered and trimmed, de novo assembled, and taxonomically classified (*7*,*8*). Analysis of the NGS results identified a complete West Nile virus genome (11,060 nt). To confirm this result, a novel RNA extraction and a reverse transcription PCR (QIAGEN, Venlo, the Netherlands) with newly developed primers were performed on the BAL sample and cell culture, followed by Sanger sequencing (*9*). The sequence obtained from the positive sample was identical to the sequence from the complete genome obtained by NGS. 

The WNV lineage 2 strain in this study, WNV-2|Belgium|2017|Antwerp (GenBank accession number: MH021189), is closely related (99.4%–99.6% nucleotide similarity) to a strain from Hungary and other Balkan countries identified between 2010 and 2015 (Figure), reinforcing the conclusion that this WNV infection was acquired in Hungary. This case illustrates that the combined use of cell cultures and NGS can be a powerful tool for identifying unknown pathogens in clinical specimens when results from routine tests are negative and the patient’s condition is undiagnosed. 

**Figure:**
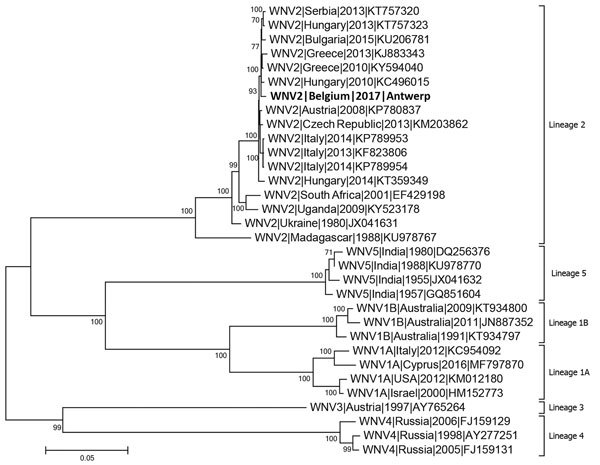
Alignments of complete WNV genome identified from patient in Belgium compared with 32 known reference strains from the 5 established WNV lineages. A phylogenetic tree was constructed using the maximum-likelihood method based on the Tamura-Nei model. Evolutionary analyses were conducted in MEGA7 (http://www.megasoftware.net). Bootstrapping was conducted with 1,000 bootstrap replicates; only bootstrap values over 70% are shown. The phylogenetic tree clusters the strain from this patient together with other WNV strains from southeastern Europe in lineage II. The patient isolate, named WNV-2|Belgium|2017|Antwerp (GenBank accession no. MH021189), is most similar to a strain from Hungary. GenBank accession numbers are provided for reference isolates. Scale bar represents genetic distance.
